# Relapsing pericarditis mimicking aortic dissection

**DOI:** 10.1093/ehjcr/ytaf111

**Published:** 2025-02-27

**Authors:** Yoshiko Eto, Nobuhiro Tahara, Yoshihiro Fukumoto

**Affiliations:** Division of Cardiovascular Medicine, Department of Medicine, Kurume University School of Medicine, 67 Asahi-machi, Kurume 830-0011, Japan; Division of Cardiovascular Medicine, Department of Medicine, Kurume University School of Medicine, 67 Asahi-machi, Kurume 830-0011, Japan; Division of Cardiovascular Medicine, Department of Medicine, Kurume University School of Medicine, 67 Asahi-machi, Kurume 830-0011, Japan

A 73-year-old woman with heart failure symptoms was admitted to our hospital. On admission, cardiac and mediastinal silhouettes were enlarged on a chest roentgenogram (*Panel A*). The patient was afebrile with a heart rate of 118 b.p.m. Electrocardiography indicated ST segment elevations in all leads (*Panel C*). Transthoracic echocardiography showed trivial regurgitation from a normal trileaflet aortic valve, hyperkinetic motion of the left ventricle, and pericardial effusion (*Panel D*, arrows). Computed tomography with contrast media revealed aortic dissection-like features around the ascending aorta (*Panels E* and *F*, arrowheads) and pleural and pericardial effusion (*Panel G*, arrowheads). Although she was haemodynamically stable, pericardiocentesis was performed to rule out aortic dissection. Non-bloody serosanguinous fluid was obtained and contained no malignant cell. On detailed interview, the patient reported two histories of pericarditis at the ages of 71 and 72. Therefore, she was diagnosed with relapsing pericarditis. After the initiation of oral corticosteroid therapy, the pleural, pericardial, and mediastinal effusion disappeared. Subsequently, cardiac and mediastinal silhouettes got smaller on a chest roentgenogram (*Panel B*). The patient was discharged with no demonstrable complications.

**Figure:**
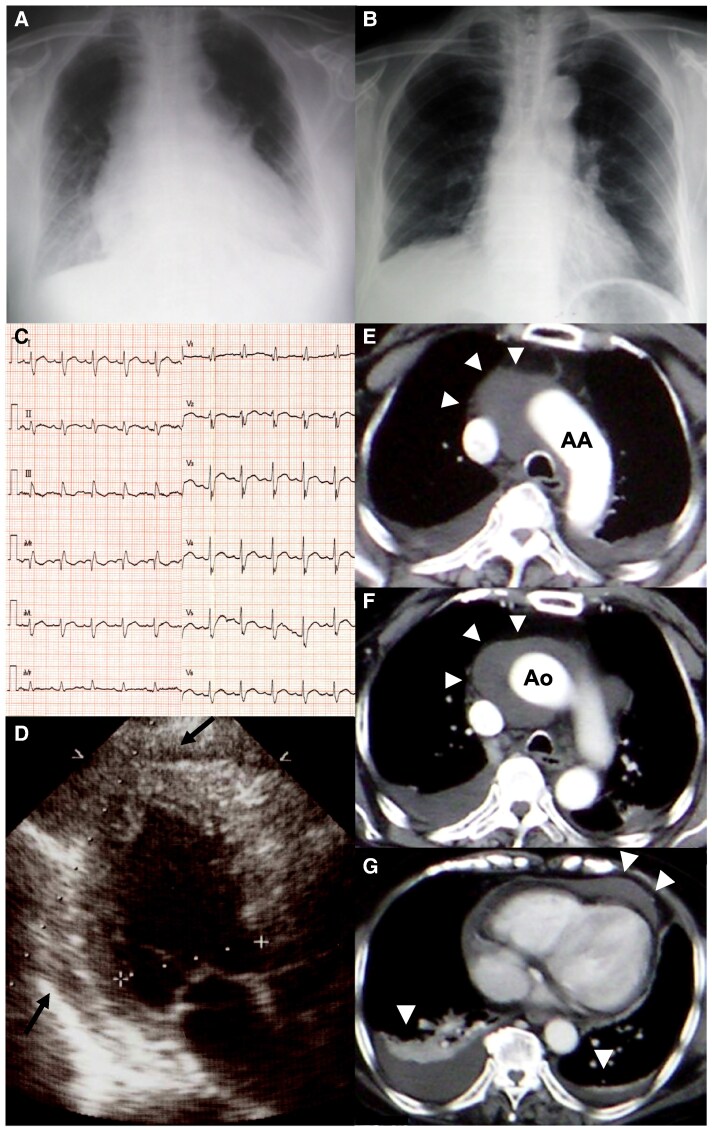
(*A*) Chest roentgenogram on admission. (*B*) Following chest roentgenogram after pericardiocentesis and the initiation of oral corticosteroid therapy. (*C*) Electrocardiogram indicating ST segment elevations in all leads. (*D*) Transthoracic echocardiogram showing pericardial effusion. (*E–G*) Enhanced computed tomography demonstrating aortic dissection-like features around the ascending aorta and pleural and pericardial effusion (arrowheads). AA, aortic arch; Ao, ascending aorta.

This is a rare case of relapsing pericarditis manifested mainly by massive effusion around the ascending aortic root, prompting initial concern of aortic dissection. Awareness and careful assessment are required to exclude an acute aortic syndrome in such patient. Physicians should carefully analyse their clinical findings.

**Consent:** The patient gave consent to appear in the publication in accordance with the COPE guidelines.

**Funding**: None declared.

## Data Availability

The data underlying this article will be shared on reasonable request to the corresponding author.

